# A DNA barcode-based survey of wild urban bees in the Loire Valley, France

**DOI:** 10.1038/s41598-021-83631-0

**Published:** 2021-02-26

**Authors:** Irene Villalta, Romain Ledet, Mathilde Baude, David Genoud, Christophe Bouget, Maxime Cornillon, Sébastien Moreau, Béatrice Courtial, Carlos Lopez-Vaamonde

**Affiliations:** 1 IRBI, UMR 7261, CNRS, Université de Tours, Tours, France; 2 INRAE USC 1328, LBLGC EA 1207, Université d’Orléans, Orléans, France; 3 Domaine Bellevue 2, Arzens, France; 4 INRAE, UR EFNO, Nogent-sur-Vernisson, France; 5 CETU Innophyt, Université de Tours, Tours, France; 6 INRAE, URZF, Orléans, France

**Keywords:** Biodiversity, Conservation biology, Ecological genetics, Haplotypes

## Abstract

The current decline of wild bees puts important ecosystem services such as pollination at risk. Both inventory and monitoring programs are needed to understand the causes of wild bee decline. Effective insect monitoring relies on both mass-trapping methods coupled with rapid and accurate identifications. Identifying wild bees using only morphology can be challenging, in particular, specimens from mass-trapped samples which are often in poor condition. We generated DNA barcodes for 2931 specimens representing 157 species (156 named and one unnamed species) and 28 genera. Automated cluster delineation reveals 172 BINs (Barcodes Index Numbers). A total of 36 species (22.93%) were found in highly urbanized areas. The majority of specimens, representing 96.17% of the species barcoded form reciprocally exclusive groups, allowing their unambiguous identification. This includes several closely related species notoriously difficult to identify. A total of 137 species (87.26%) show a “one-to-one” match between a named species and the BIN assignment. Fourteen species (8.92%) show deep conspecific lineages with no apparent morphological differentiation. Only two species pairs shared the same BIN making their identification with DNA barcodes alone uncertain. Therefore, our DNA barcoding reference library allows reliable identification by non-experts for the vast majority of wild bee species in the Loire Valley.

## Introduction

Long-term monitoring programs have documented a sharp decline of insects^1–5^. The loss of insect pollinators is particularly worrying because of its potential negative ecological and economic consequences^6–8^. Land use change has been shown to be a major factor involved in the loss of worldwide pollinator populations^9^. Indeed, intensive agriculture has led to the loss of ecological niches for a number of pollinator species, to which are added the adverse effects of pesticide uses^10^. Through the expansion of impervious surfaces, urbanization is also associated with pollinator decline^11,12^, although some urban green areas such as residential and community gardens, if properly managed, can constitute important refuges for wild bees^13–15^. This factor and the higher temperatures associated with rapid global warming are accelerating the decline of pollinators worldwide^16,17^. This scenario has fostered the idea of considering urban areas as potential refuges for pollinators^18,19^. Consequently, a growing number of studies on urban ecology have emerged describing population dynamics of wild bees in urban areas^19,20^ due to their pivotal importance. In addition, citizen science has been successfully shown to be an efficient way of monitoring urban bees^21–24^. However, the accurate identification at species level for several bee genera requires advanced taxonomic knowledge, which is limited to a few experts or even not available in many countries. This taxonomic impediment is slowly being overcome through the use of traditional DNA barcoding^25–28^ or more recently developed high-throughput DNA barcoding^29–33^. Indeed, *cox1* (cytochrome *c* oxidase subunit I gene) barcodes have been shown to distinguish between bees difficult to identify due to minor morphological differences^34–36^ or even cryptic bee species^37^.

The accuracy of DNA-based identifications depends on the completeness of DNA barcoding reference libraries. However, only a few DNA barcode reference libraries have been developed for wild bee national faunas, including Ireland^38^; Germany^27^, Canada^25,39^ and Chile^28^ as well as some regional faunas^26,40,41^. In addition, identification accuracy depends also on the complete characterization of intraspecific variability^42–44^.

France has a rich wild bee fauna with over 955 species recorded^45^, for the whole country but relatively few DNA barcodes of French bees have been published^46–48^. Our study is the first major contribution to establish a DNA barcoding reference library for French wild bees.

We focused our sampling in central France, a region where over 180 species of bees have been recorded (unpublished data, Christian Cocquempot personal communication, 2020). Here we present 2931 barcodes of 157 wild bee species collected at 29 urban and peri-urban sites in three major cities along the Loire Valley.

## Results

### Taxon sampling and DNA sequencing success

**Figure 1:**
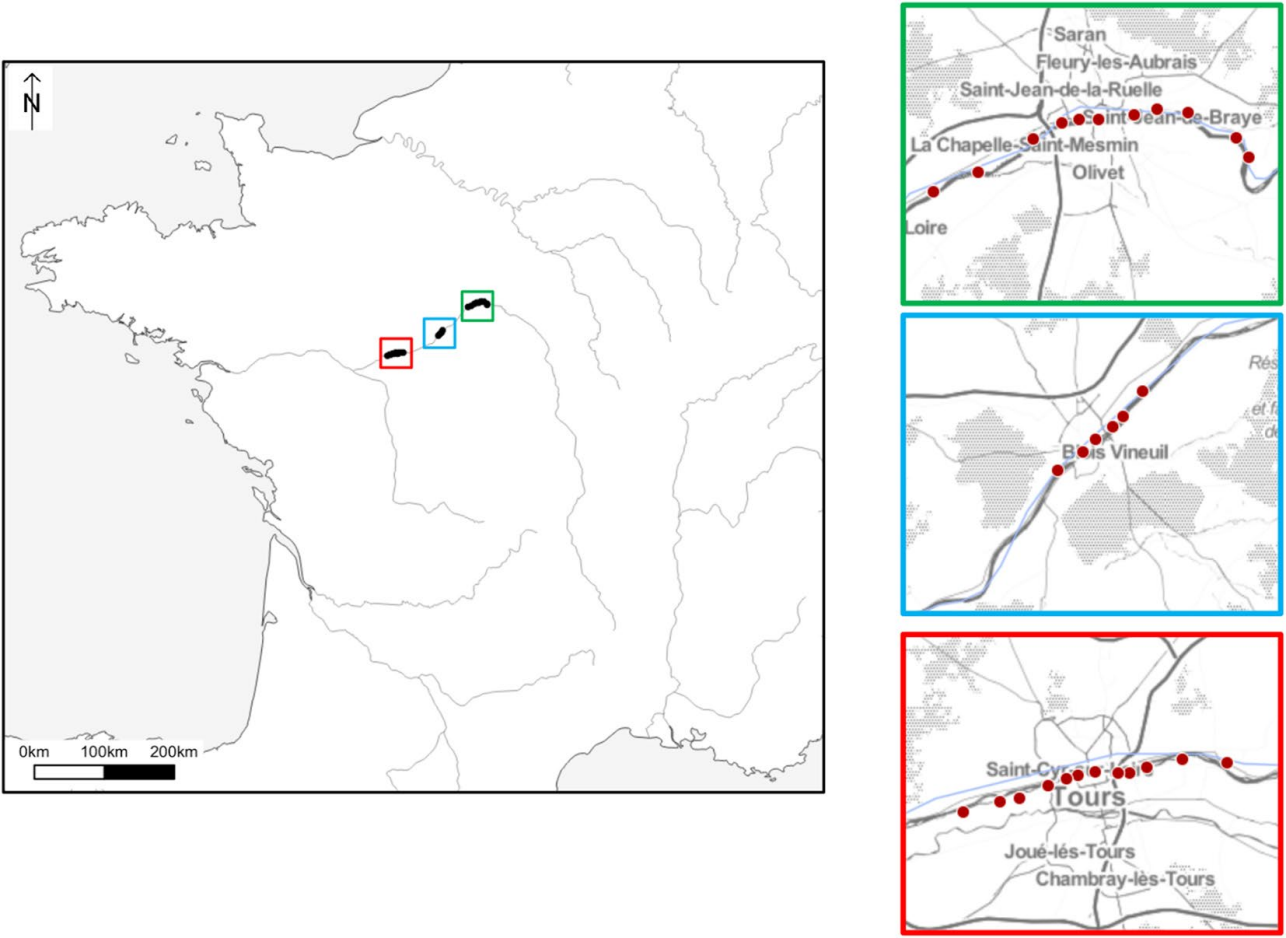
Map locations for all sampling from the three main regions in the Loire Valley. The software QGIS v.3.8 was used to represent sampling sites (http://www.qgis.org).

A total of 3532 bee specimens were collected over the 2 years of survey; 3057 bees were collected with pan-traps and 475 were collected along transects in 29 sites located in three French cities (Tours, Orléans and Blois) (Fig. 1, Table S1).

Of the 3532 specimens collected 2931 were successfully barcoded (82.98%) (Table S2). The overall success rate varied depending on the sequencing method, Sanger versus Single-Molecule Real-Time sequencing (SMRT^49^) and the primers used. Out of the 252 samples analysed by Sanger sequencing, we obtained a higher barcoding success rate using newly designed primers (133 sequences out of 188 specimens, i.e. 70.74%) (Table S3) than with the traditional Folmer primers (15 sequences out of 64 specimens, i.e. 23.43%). We obtained full DNA barcodes for 13 species whereas six species were represented only by short sequences (≥ 300 bp and < 500 bp). Twenty-seven out of 148 sequences were less than 500 bp long. Of the 3350 specimens processed using PacBio Sequel platform (Pacific Biosciences, Menlo Park, CA, USA) by SMRT sequencing, 2783 specimens (83.07%) yielded a barcode sequence > 300 bp. Six out of 2783 were less than 500 bp. No barcodes could be obtained for 410 additional samples and 157 samples (4.69%) appeared to be cross contaminated. Sequencing failure was not homogeneous across families, and a few species presented greater amplification problems, especially among the genera *Andrena*, *Hylaeus* and *Dasypoda*.

### Species identification and BIN assignment

**Figure 2:**
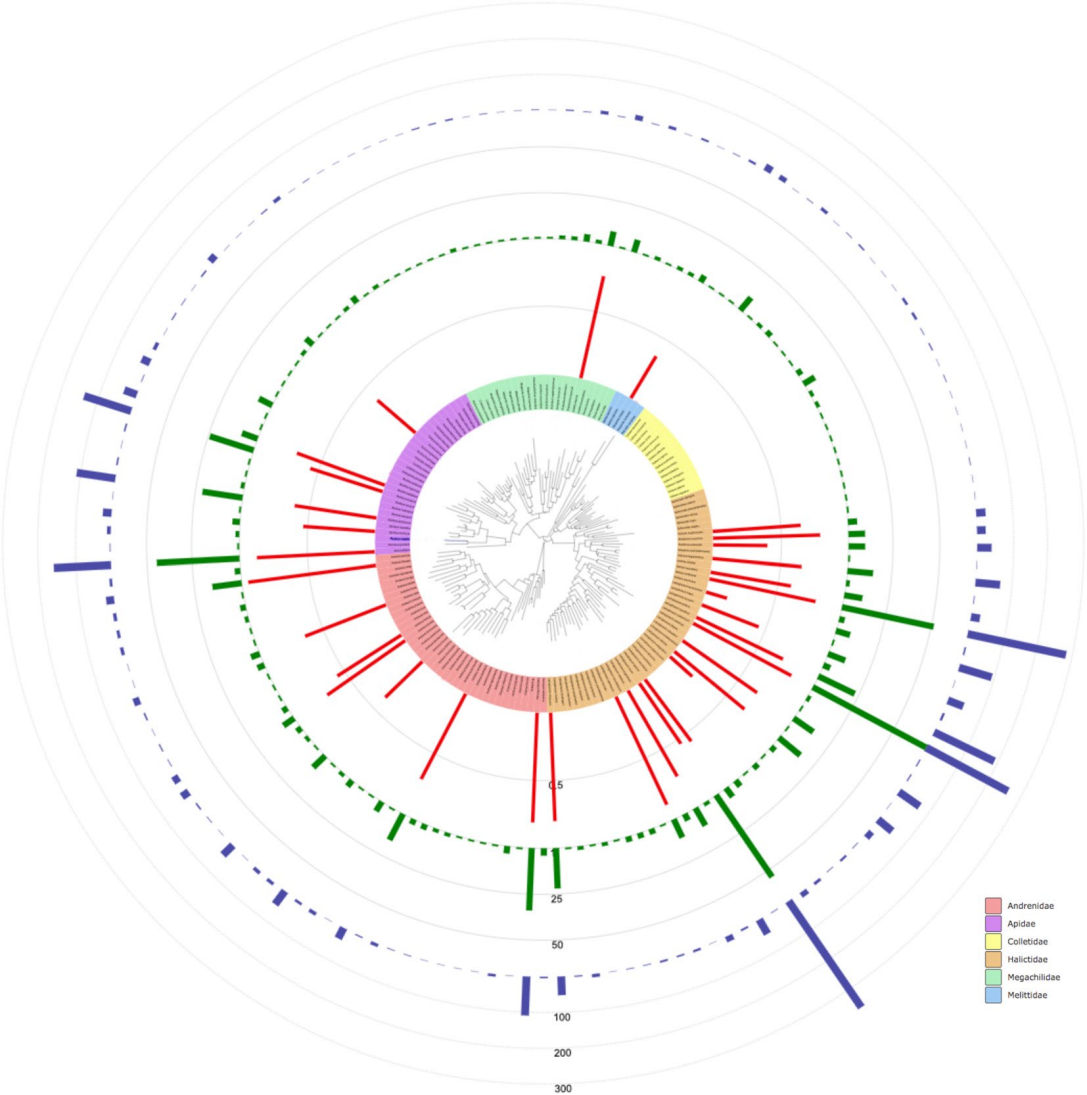
Approximately Maximum-Likelihood tree of species found in our study. Species are color-coded by family. Bars represent the abundance of each species (blue), the number of haplotypes (green) and the values of haplotype diversity only for species with n > 10 (red).

Our integrative analyses combining morphology and DNA barcodes, identified a total of 157 species out of which 156 are described valid species belonging to six families (Andrenidae, Apidae, Colletidae, Halictidae, Megachilidae, Melittidae) and 28 genera (Tables 1, S4, Fig. 2). In addition, we found one specimen (MB00149) of an unnamed *Andrena* species whose BIN was new to BOLD. Seventy species (44.6%) found in our study had not been reported before within the available regional species inventories. For instance, our barcode of *Andrena avara liturata* (Warncke) represent a first record for the studied area, and a new BIN in BOLD (Barcode of Life Data).

**Table 1:** Number of barcoded records, genera and species collected for the six bee families in our sampling.

Family	Barcoded records	Genera	Species
Andrenidae	430	2	38
Apidae	579	8	29
Colletidae	44	2	15
Halictidae	1766	4	47
Megachilidae	69	9	23
Melittidae	43	3	5
Total	2931	28	157

Of the 157 species 156 were assigned to 172 BINs (results on 25 September 2020), sixteen (9.30%) of them were new to BOLD. Only one of the 157 species barcoded, did not have a BIN assigned, since its barcode was shorter than 500 bp^50^.

Out of the 157 species barcoded from our studied area 137 (87.26%) had already been barcoded from Germany (Table S4).

A total of 36 species (22.93%) were found in highly urbanized areas (i.e. city centers).

Halictidae accounted for most of the specimens, representing just over 60% of the total specimens collected and 14.3% of genera. The most diverse families in terms of number species were Halictidae and Andrenidae, accounting for 30% and 24% of species, respectively. Only five species (3.18%) of Melittidae were observed (Tables 1, S4).

The majority of species (96.17%) had their own unique BIN or were assigned to several BINs that formed single clades allowing unambiguous identification based on DNA barcodes; 48 species were represented by a single record. The number of specimens sequenced per BIN ranged from 1 to 360 (for *Lasioglossum morio* (Fabricius)), averaging 17 specimens per BIN. Sixty-one BINs were represented by a single individual (singletons).

Multiple BINS were associated with 14 species, with up to three BINS for some, including *Andrena helvola* (Linnaeus), *Anthophora plumipes* (Pallas), *Lasioglossum laticeps* (Schenck) and *L. villosulum* (Kirby) (Tables 2, S4).

**Table 2:** Sampled bee species barcodes assigned to multiple BINs by the BOLD system database.

Family	Species	Author	N	BIN name (N specimens per BIN)
Andrenidae	*Andrena bicolor*	Fabricius, 1775	7	BOLD:AAD0134(5) BOLD:AAD0135(1)
Andrenidae	*Andrena helvola*	Linnaeus, 1758	19	BOLD:ABU9089(1) BOLD:ACY0380(7) BOLD:ADZ3664(11)
Andrenidae	*Andrena lagopus*	Latreille, 1809	2	BOLD:AAK0222(1) BOLD:ACC2245(1)
Andrenidae	*Panurgus calcaratus*	Scopoli, 1763	4	BOLD:AAE3229(3) BOLD:AED3388(1)
Apidae	*Anthophora plumipes*	Pallas, 1772	10	BOLD:AAF1671(2) BOLD:AAF1672(7) BOLD:AAZ7403(1)
Halictidae	*Halictus langobardicus*	Blüthgen, 1944	69	BOLD:AAD5869(68) BOLD:ACE9465(1)
Halictidae	*Halictus maculatus*	Smith, 1848	25	BOLD:AAY5383(22) BOLD:ACH4344(3)
Halictidae	*Lasioglossum laticeps*	Schenck, 1868	51	BOLD:AAY5433(46) BOLD:ADZ4826(1) BOLD:ADZ6624(2)
Halictidae	*Lasioglossum minutissimum*	Kirby, 1802	5	BOLD:AAI1289(1) BOLD:ACQ8646(4)
Halictidae	*Lasioglossum subhirtum*	Lepeletier, 1841	3	BOLD:ADM2541(2) BOLD:ADZ3360(1)
Halictidae	*Lasioglossum villosulum*	Kirby, 1802	52	BOLD:AAC2460(5) BOLD:AAC2461(46) BOLD:AEC1752(1)
Halictidae	*Lasioglossum zonulum*	Smith, 1848	46	BOLD:AAB3147(41) BOLD:AAB3148(5)
Megachilidae	*Osmia bicornis*	Linnaeus, 1758	8	BOLD:AAD6282(7) BOLD:ADZ8010(1)
Melittidae	*Dasypoda hirtipes*	Fabricius, 1793	20	BOLD:AAI9629(19) BOLD:AEC2767(1)

Two species pairs share the same BIN: *Andrena carantonica* (Pérez) and *A. trimmerana* (Kirby) (BOLD:AAD2472) and *Halictus simplex* (Blüthgen) and *H. langobardicus* (Blüthgen) (BOLD:AAD5869). The identification of those four species based on DNA barcode data is therefore uncertain. One species barcoded in our study (*Lasioglossum mediterraneum* (Blüthgen)) was new to BOLD but the sequence was too short to have a BIN assigned. In addition, one unidentified individual in the genus *Andrena* (MB00149) was assigned to a new BIN (BOLD:ADZ3755) to BOLD. This new BIN remains without species identification pending of collection and analysis of more individuals.

### Barcode gap and haplotype diversity

**Figure 3:**
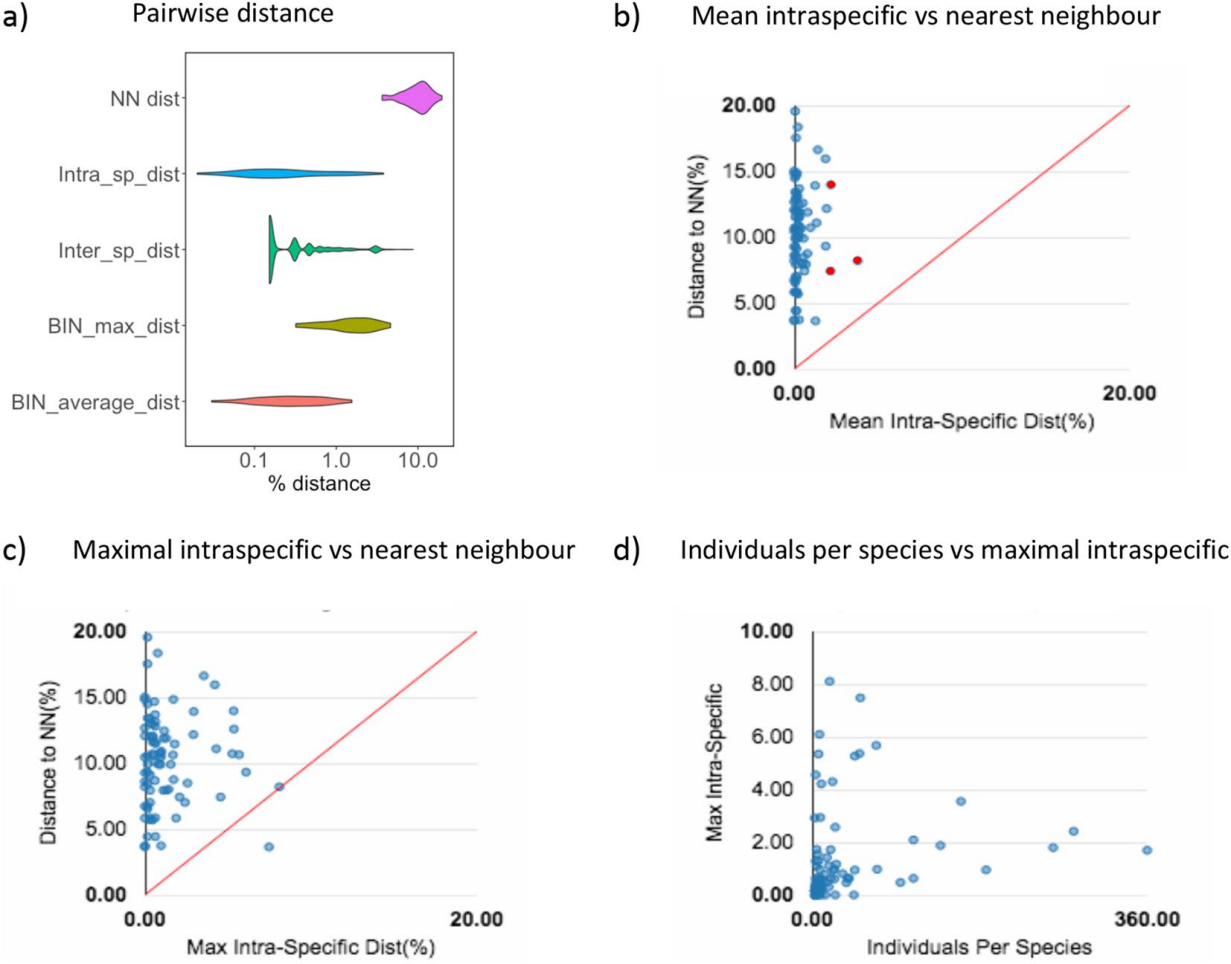
Kimura 2 Parameter distances for species with a number of records n ≥ 3. (**a**) Violin plot representing Nearest-neighbor (NN), Mean Intra and Inter specific genetic distances and BIN maximal and average distances; (**b**) Mean intra-specific distances vs the minimum inter-specific distances, species with intra-specific distances over 2% are depicted in red; (**c**) Maximal intra-specific distances *vs*. the minimum inter-specific distances; (**d**) Number of individuals in each species against their max intra-specific distances.

The average genetic distance within species and genera in the dataset were 0.23% and 15.70% respectively (Table 3) with a maximum intraspecific distance of 9.81% for *Andrena lagopus* (Table S4, Fig. 3a). Intraspecific barcode divergence averaged 0.23% whereas distance to the nearest-neighbour species averaged 9.08%. When we considered only species with a number of records n ≥ 3, intraspecific divergence exceeded 2% in three species; *Panurgus calcaratus* (Scopoli) (2 BINs), *Andrena bicolor* (Fabricius) (2 BINs) and *Andrena helvola* (Linnaeus) (3 BINs) (Table S4, Fig. 3b).

**Table 3:** Kimura 2 Parameter sequence divergence between barcode sequences at the species, genus and family level.

Label	n	Taxa	Comparisons	Min Dist (%)	Mean Dist (%)	Max Dist (%)	SE Dist (%)
Within Species	2883	109	210,415	0.00	0.23	9.81	0.00
Within Genus	2753	20	836,587	1.24	15.70	50.00	0.00
Within Family	2931	6	775,254	8.56	18.12	34.38	0.00

The mean intraspecific distance (n ≥ 3) distribution ranged from 0 to 3.78% and overlapped slightly with the distance to the nearest neighbour distribution (3.64–19.56%) (Fig. 3a,b). However, nearest-neighbour distances were on average more than tenfold higher than maximum intraspecific distances (Fig. 3a,c). *Lasioglossum villosulum* maximum intraspecific distance values were higher than their nearest-neighbour distance. Conversely, K2P distances to the nearest neighbour < 2% were obtained for two species pairs: *Andrena trimmerana/A. carantonica* and *Halictus langobardicus/H. simplex* (Table S4). Maximum intraspecific divergence was not correlated with the number of individuals sampled per species (R = 0.174, P = 0.113 (Fig. 3d).

Our data set contained 787 unique haplotypes with an average of five haplotypes per species and a maximum of 70 haplotypes for *Lasioglossum malachurum* (Kirby). We found variable patterns of haplotype diversity among the species (Table S4). The number of haplotypes per species ranged from one to 70, and the values of haplotype diversity from 0 to 0.95. *Andrena flavipes* (Panzer) had the highest haplotype diversity value, followed by *Lasioglossum sabulosum* (Warncke), *Apis mellifera* (Linnaeus) and *Lasioglossum malachurum* (Table S4). When we considered species with more than ten individuals, the number of samples collected per species was highly correlated with the number of haplotypes found (R = 0.898, P = 1.114e − 13), but not with the values of haplotype diversity (R = 0.228, P = 0.181) (Fig. 2).

## Discussion

DNA barcoding allows the unambiguous identification of over 96.17% of the wild bee species found in our study. Among them species that are notoriously difficult to identify morphologically like: *Bombus terrestris/lucorum* complex^51^.

Our barcode analysis revealed one interesting *Andrena* individual collected in the national reserve of Saint Mesmin, which was assigned to a BIN new to BOLD, and remains unnamed at the species level. By sequence homology, this individual belongs to the ‘*Andrena bicolor*’ group, and its nearest neighbour is *Andrena allosa* (Warncke). The *Andrena bicolor* species group has been recently revised to clarify the status of several alpine species^52^, the results suggest the existence of a substantial cryptic diversity in southern European *Andrena* (*Euandrena*). In the present study in parallel to the new BIN attributed to this specimen (BOLD:ADZ3755), BINs BOLD:AAD0134 and BOLD:AAD0135 are also from the ‘*Andrena bicolor*’ group. Considering that two of these three BINs are represented by only one individual is very difficult to morphologically determine if these BINs represent different species or if they are representatives of subspecies of the ‘*Andrena bicolor*’ group that are not well represented so far in the BOLD database. Our specimen could indeed represent a new species or subspecies within this cryptic complex, but more specimens are needed to assess its taxonomic status.

The two species pairs that shared a BIN *Andrena trimmerana/A. carantonica* and *Halictus langobardicus/H. simplex* are morphologically very similar. Indeed, females of *Halictus simplex*/*langobardicus*/*compressus* complex are morphologically indistinguishable^53^. Similarly, females of both *Andrena carantonica* and* A. trimmerana* show no morphological differentiation. The BIN shared by both *Andrena carantonica* and *A. trimmerana* also contains specimens of *A. scotica* (Perkins) and *A. spinigera* (Kirbi). A taxonomic revision of these species is needed to clarify their status within *Andrena*. The morphological identification of specimens of these difficult groups must be done by expert taxonomists with access to reference collections including types, and comparing specimens from several geographical origins.

More than half of the bees sampled in our study, and includes the most abundant species, belong to the Halictidae (Table 1, Fig. 2). Other studies have reported similar results with halictid bees representing the dominant group between 52.7 and 98.7% of records sampled in different countries and habitats^54–60^. Halictidae has also been the most abundant family in bee monitoring studies of other French cities^11,12,61^. Pan-trapping has previously been associated with an excessive catching of halictids compared to other families collected^62^. Although we have complemented our sampling with netting, the taxonomic overrepresentation of our sampling towards halictids (that are ground nesting bees) is likely to be caused by pan-trapping.

Sequencing failure for some bee taxa has been reported especially among *Andrena*^38^ and *Hylaeus* species^63^. They have been attributed with inefficient primer annealing in the case of *Andrena* and with the presence of heteroplasmy for *Hylaeus* species^63^. We have sequenced only a few samples with Sanger sequencing methods, and individuals that failed to amplify the barcode sequence were repeated by SMRT sequencing methods. SMRT barcoding methodology eliminates the potential sequencing issues associated with the amplification of multiple mitochondrial copies or with the amplification of nuclear mitochondrial pseudogenes (numts). However, the presence of numts could contribute to failures in sequencing or the sequencing of contaminants. Sampling, storage conditions and DNA extraction methods can be at the origin of a number of sequencing problems^64^. Our pan-trapping involved bees being kept in soapy water for up to 4 days. This could favour DNA degradation and cross-contamination of samples affecting the rate of DNA sequencing success (82%) compared to the results obtained on DNA amplification of swept individuals (90%).

Our data shows a marked difference between mean intraspecific (0.23%) and interspecific (15.70%) genetic divergence indicating the existence of a species barcode gap^65^ in our dataset (Table 3). This low average intraspecific divergence has also been reported in Canadian wild bees^26^.

Here we report an 83.07% success rate using SMRT sequencing compared to 70.74% with Sanger sequencing using our newly designed primers. Sanger sequencing remains the gold standard in terms of quality and reliability for small projects of a few hundred samples. However, Sequel platform can greatly reduce sequencing costs and be more competitive for projects with thousands of samples^49^.

Our analyses revealed 14 species (9%) with multiple BINs (Table 2). A barcoding study on German bees found a similar level (11%) of species with multiple BINs^27^. Among the 14 species that show deep mitochondrial splits, most belonged to *Andrena* (3 spp.) and *Lasioglossum* (5 spp.), the latter being the most species-rich genus of bees worldwide^66^. The identification of *Andrena* species is considered challenging due to the great diversity of the genus and the complex *Andrena* subgeneric keys^67,68^, which makes the identification of species represented by single individuals very difficult. Cases of cryptic diversity and/or deep intraspecific divergences have been suggested for this genus^52^ and the taxonomic status of several species remains uncertain^27,69^.

We have reported five new BINS within the *Lasioglossum* genus*. Lasioglossum* is considered morphologically homogeneous and relatively difficult to identify^35,70,71^. Our study has found three BINs within the *Lasioglossum villosulum* complex. The species complex formed up to five BINS has been the subject of a recent taxonomic revision^72^ which has resurrected *Lasioglossum medinai* (Vachal) and *L. berberum* (Benoist). The higher values of maximum intra-specific distances compared to the distance to the nearest neighbour suggest the existence of cryptic diversity for the species complex.

A genomic approach^41,73^ is necessary to further investigate the deep intraspecific DNA barcode splits (Table 2) observed in our study.

The French wild bee fauna has a high overlap with the German fauna specially for central European species. However, most of the Mediterranean wild bee fauna remains to be barcoded. We have found a relatively high species richness of bees in urban areas with 36 species occurring in city centers. Other studies have also found similar levels of species richness in highly urbanized areas^20^. In the periurban areas we found a richer community of wild bees, one particularly interesting species we found is *Megachile genalis* (Morawitz), which occurs in marshy meadows rich in both *Carduus* and *Cirsium* (Asteraceae) plants. It nests in the stems of those plants and feeding preferentially on Asteraceae as well as other plants with wide stems such as *Oenanthe* and *Angelica* (Apiaceae).

Our studied fauna of the Loire Valley being in central France has few Mediterranean species and therefore a high overlap with that of Germany (87.26%). Although many of the species occurring in the Loire valley are already barcoded from Germany the French barcodes will help to fully characterize the intraspecific variability of European wild bees.

## Conclusion

Our DNA-based survey represents a major contribution to the barcoding of European wild bees^27,31,38^ both at the inter and intraspecific levels. Our records were assigned to 172 BINs, 16 of which were new to BOLD. Of the 157 species reported in this study, only 67 of them had previously been barcoded from France.

DNA barcoding using Sequel platform represents a helpful tool to process large numbers of bees to obtain not only species richness but also abundance data for further ecological analyses.

Our study revealed fourteen species with multiple BINs without morphological differentiation suggesting the existence of cryptic diversity. We have found a relatively high species richness of bees in urban areas. Our DNA barcode reference library will help to streamline the identification of wild bees and assess the impact of anthropogenic disturbances such as urbanization. Finally, it is a major contribution to the ultimate goal of completing the barcoding of all wild bee European fauna in a short time framework.

## Methods

### Field sampling

Wild bees (3532) were collected in 29 sites located in three cities of central France along alluvial areas of the Loire valley (Fig. 1, Table S1). The 30-year annual average temperature in our sampling area is 11.1 °C with a minimum of 3.4 °C in January and a maximum of 19.1 °C in July. Average precipitation is 54.2 mm with a maximum of 64.3 mm in May (https://fr.climate-data.org).

The sampled material was collected over 2 years: in 2017 from April 11th till July 19th and in 2018 from April 23rd to July 17th (including *Apis mellifera* Linnaeus).

Bees were collected using a combination of pan-trapping and net sampling^74^.

*Pan-traps* Each of the 29 monitoring sites were equipped with yellow, blue and white UV-reflecting coloured plastic bowls (120 mm diameter × 120 mm height) and filled with 500 ml of soapy water. The pan-traps were arranged in triplets; each triplet consisted of the three coloured bowls fixed to a wooden stick at the height of the vegetation^75^. In the 2017 season, two triplets were installed 100 m apart to each other at each site for an exposure time of 48 h, repeated for five trapping sessions. In the 2018 season, the trapping time and the number of triplets were increased to enhance the sampling effort: three triplets spaced at 50 m apart were placed at each sampling site for an exposure time of 96 h, repeated for five sessions. At the end of each trapping session, the traps were lifted and filtered. Bees were conserved in 96° ethanol and stored in the freezer at − 20 °C until identification.

*Net sampling* A butterfly net was used to ensure the sampling of individuals from genera such as *Bombus* and *Colletes*, known to be inefficiently captured with pan-traps^74,75^. Ten-minute capture sessions took place on 100 m transects, which corresponded to the distance between traps. We captured as many flower foraging individuals as possible during these sessions. In 2018, five net-sweeping sessions took place in each of the 29 sampling sites.

All 3532 collected bee specimens were pinned and imaged. We stored one hind leg of each individual on 96 well plates with 30 μL of 96% ethanol in each well for DNA barcoding.

Morphological identifications were performed based on keys and published descriptions^69,76–79^.

### Laboratory procedures and analyses of DNA barcoding data

Two methods were used to generate DNA barcodes.

Of the 214 samples 148 were DNA barcoded using standard Sanger sequencing at URZF INRAE Orléans. Total DNA was extracted from one hind leg using the NucleoSpin Tissue XS Genomic DNA Purification kit (Macherey–Nagel Inc.), following the manufacturer’s protocol. The barcode fragment of mitochondrial cytochrome *c* oxidase subunit I gene was first targeted with the standard primers LCO and HCO^80^. We ran PCR using Dream Taq Green DNA polymerase (Thermo Fisher Scientific) with a final concentration of 2.5 mM MgCl_2_ and 1 µM for each primer and the following program: a starting denaturation step at 95° for 3 min, followed by five cycles of 95 °C for 30 s, 47 °C for 40 s and 72 °C for 60 s, and 40 cycles of 95 °C for 30 s, 52 °C for 40 s and 72° for 60 s, and a final extension step at 72° for 5 min. To amplify problematic taxa, two new primers were designed (Table S3) and used in combination with the following PCR program: a starting denaturation step at 95° for 3 min, followed by ten cycles of 95 °C for 30 s, 59–50 °C (− 1 °C per cycle) for 40 s and 72 °C for 60 s, and 45 cycles of 95 °C for 30 s, 56 °C for 40 s and 72° for 60 s, and a final extension step at 72 °C for 5 min. Amplification was confirmed by agarose gel electrophoresis. PCR products were subsequently cleaned using the NucleoFast 96 PCR kit (Macherey–Nagel, Düren, Germany) and sequenced in both directions by the Sanger method using the ABI Prism BigDye Terminator v3.1 Cycle Sequencing Kit (Thermo Fisher Scientific). Sequencing reactions were purified by ethanol precipitation, loaded on an Applied Biosystems 3500 Genetic Analyzer (Thermo Fisher Scientific). We used CodonCode (CodonCode Corporation, Centerville, MA) for primers trimming, contig assembly and sequence editing. Sequence alignment was straightforward in the absence of indels and the sequences along with corresponding trace files were uploaded to the Barcode of Life Data Systems (BOLD) (http://www.barcodinglife.org)^81^.

A total of 3350 tissue samples (hind legs) were DNA barcoded using single-molecule real-time sequencing (SMRT^49^) in the PacBio Sequel platform (Pacific Biosciences, Menlo Park, CA, USA) at the Canadian Centre for DNA Barcoding (CCDB), in the University of Guelph, Ontario, Canada).

Barcode DNA sequences were aligned in Geneious v.8.0.3 (www.geneious.com) through the MAFFT alignment tool^82^ to verify the correct *cox1* codon translation into proteins and check for potential presence of numts by looking for stop codons, insertions and deletions, errors and inconsistencies. We edited SEQUEL sequences with minor errors caused by the addition or deletion of a single nucleotide that initiated a reading frame shift and the introduction of stop codons.

All sequences, along with the voucher data, images, and trace files, are deposited in BOLD and the sequences are deposited in GenBank (Table S2). All data are available from the BOLD database, 10.5883/DS-BEECOI.

We used the Barcode Index Number system (BIN)^50^ to delineate species using DNA barcode data. BINs were assigned automatically to each record with a barcode longer than 500 bp based on the Refined Single Linkage (RESL) algorithm in BOLD 4.

Sequence alignment generated by the amino acid-based (HMM) BOLD aligner was used to construct a Kimura 2 parameter^83^ (K2P) Neighbour‐joining tree (File S1) to provide a graphic representation of the species divergence and calculate genetic intra and interspecific distances through the BOLD analytical tools support^81^. We constructed an approximately Maximum-Likelihood tree using Fast Tree2 with default settings^84^ in Geneious v.8.0.3 (www.geneious.com) which was then used to represent species abundance, number of haplotypes and haplotype diversity values with the ITOL tree viewer^85^.

We used R ggplot2^86^ to represent genetic intra and interspecific distances. Species boundaries were verified by comparing the maximum intraspecific distance and the distance to the nearest phylogenetic neighbour in the data set. The software DNAsp^87^ was used to calculate the number of haplotypes per species and the values of haplotype diversity^88^ when applicable (Table S4).

## Supplementary Information

Supplementary Legends.

Supplementary Information 1.

Supplementary Information 2.

Supplementary Table S1.

Supplementary Table S2.

Supplementary Table S3.

Supplementary Table S4.

Supplementary Table S5.

## Data Availability

Dataset title: POLLEN DNA barcode reference library. Resource link: 10.5883/DS-BEECOI. Number of data sets: (1) Data set name: DS-BEECOI. Data format: xml, tsv, fasta, ab1. Description: The POLLEN library dataset could be accessed through the Workbench platform of BOLD, and all data files can be downloaded from the BOLD public portal in different formats depending on the dataset type. All records are also accessible within BOLD, using the search function of the database. An excel spreadsheet containing the library dataset information is included (Table S5). Similarly, the fasta sequences of dataset barcodes are included as File S2. We compiled a checklist of the 157 species of bees found in our study (CL-BEE37 in BOLD) following the higher classification of TAXREF taxonomic repository.
