# Supplementary material for: A DNA barcode-based survey of wild urban bees in the Loire Valley, France
Source: Sci Rep. 2021 Feb 26;11:4770. doi: 10.1038/s41598-021-83631-0 (PMC7910470; doi:10.1038/s41598-021-83631-0)
Supplement: Supplementary file 1 — Supplementary Legends. [file 41598_2021_83631_MOESM1_ESM.docx]

**Supporting information**

S1 Table: GPS coordinates of sample collection sites.

S2 Table. GenBank accession numbers of wild bee barcodes. List of voucher specimens with sample ID, sequencing method used, BIN and GenBank accession numbers.

S3 Table: Primer sequences designed for this study.

S4 Table. Wild bee species barcoding summary. Family name, species name, species description author, number of specimens, species found in city centers, mean intraspecific distance, maximum intraspecific distance, barcode index numbers (BIN) haplotype diversity values found in our study and database with distribution, Barcode Index Number (BIN) attributed to species (number of records attributed to the BIN), distance to nearest neighbour, nearest neighbour species name, number of haplotypes, haplotype diversity, haplotype diversity variance found in our study, total number of records in France, total number of BINs attributed to this species in France, total number of records in BOLD database, total number of BINs attributed to this species in BOLD database, number of countries sharing this species record, countries with barcodes for this species, BINs attributed to the species (number of records attributed to each BIN), mean intraspecific distance, maximal intraspecific distance, nearest neighbour species distance in BOLD database. Distances based on Kimura 2-P model, country codes follow ISO 3166-1.

S5 Table. Record details. Spreadsheet with detailed information about collected specimens stored in BOLD webpage.

S1 File. Kimura 2 parameter (K2P) Neighbor‐joining tree. Neighbor-joining tree generated under the Kimura 2-parameter (K2P) nucleotide substitution model for the 157 wild bee species. For each individual, the BOLD sample ID code is followed by the scientific name and the BIN attributed.

S2 File. Fasta sequences of dataset barcodes.
